# The suitability of outing frequency as a definition of hikikomori (prolonged social withdrawal)

**DOI:** 10.3389/fpsyt.2023.1027498

**Published:** 2023-03-16

**Authors:** Shunsuke Nonaka, Motohiro Sakai

**Affiliations:** ^1^School of Child Psychology, Tokyo Future University, Tokyo, Japan; ^2^Faculty of Education, University of Miyazaki, Miyazaki, Japan

**Keywords:** hikikomori (social withdrawal), outing, definition, frequency, interpersonal interaction

## Abstract

Recent research has employed outing frequency, referred to in this study as one’s frequency of going out of the home, as a condition to define and determine the severity of *hikikomori* (prolonged social withdrawal). However, there is little definitive evidence on this topic. Furthermore, compared to the previous definition, it is unclear how the scope of hikikomori included in the proposed condition differs. This study aimed to clarify the relationship between hikikomori tendencies and the frequency and quality of outings to bridge this gap in research.

**Methods:**

Data included 397 self-rated online samples, 72 self-rated offline samples, and 784 parent-rated samples. Quantitative and qualitative indicators of outings and subjective social functioning impairment were used in the analysis.

**Results:**

The cut-off points supported the criteria for the number of days outside the home proposed in previous studies. The results revealed that the outing frequency condition excluded about 14.5–20.6% of those previously considered to have hikikomori. Logistic regression analysis showed that low outings with interpersonal interaction, low frequency of outings, and high subjective social functioning impairment consistently predicted hikikomori. However, outings without interpersonal interaction did not predict hikikomori.

**Conclusion:**

These results indicate that outing frequency tends to be suitable as one of the conditions for hikikomori. However, they indicate that we should also focus on the quality of outings, that is, outings with or without interpersonal interaction, to evaluate hikikomori consistently with previous findings. Further research is needed to clarify the appropriate frequency of outings to define hikikomori and determine its severity.

## 1. Introduction

Hikikomori, or prolonged social withdrawal, has attracted research attention in Japan since the 1990s and, more recently, in many other countries and regions, including America (1), Finland (2), Hong Kong (3–5), Italy (6), and Spain (7). Previous studies have reported that hikikomori cases in countries other than Japan differ in terms of family relationships (2, 8), along with higher age, a lower proportion of men, and shorter hikikomori duration than in Japan (9). This hikikomori phenomenon is accompanied by social isolation and indicates comorbidities of various psychiatric disorders, including mood, anxiety, psychotic, developmental, or personality disorders (7, 10), and poor quality of life (11, 12).

The definition of hikikomori varies between studies. The discrepancies have hindered advanced research on this topic. Many previous studies have considered conditions such as not working or attending school, not socializing outside one’s home, staying at home on most days except for solitary outings, and duration of hikikomori as the definition of hikikomori (9). The definition by the Ministry of Health, Labour and Welfare also includes these conditions (13). More recent studies have proposed the following conditions: marked social isolation in one’s home, continuous social isolation lasting at least 6 months, and significant functional impairment or distress associated with the social isolation (14, 15). In these studies, the severity of hikikomori was linked to outing frequency, here referring to as one’s frequency of going out of the home, as follows: outings on 2–3 days/week implied mild hikikomori. Going out 1 day or fewer days a week implied moderate hikikomori. Those who rarely left a single room were considered to have severe hikikomori, while those who left their homes four or more days/week did not meet the hikikomori criteria (14, 15). Thus, limited social interaction but frequent outing cases does not meet the hikikomori condition. This differs from the Ministry of Health, Labor, and Welfare’s definition, which includes outings without interpersonal interaction as hikikomori (13).

Some study findings support a relationship between outing frequency, a quantitative indicator of outings, and hikikomori tendency. For example, Yong et al. (16) reported that fewer outdoor frequencies were associated with hikikomori after adjusting for social demographics in a population study in rural Japan. A parent-rated study reported a positive correlation (*r* = 0.64) between social interaction behaviors—which show lower hikikomori—and outing frequency (17). Another study revealed that individuals with hikikomori went out less frequently per month than those who had recovered from or had not experienced hikikomori (18). Furthermore, another study suggested that the group that experienced significant changes in the frequency of outings before and after the forced isolation caused by COVID-19 showed higher depressive tendencies than the group that experienced no change in outing frequency. This finding suggests that changes in the frequency of outings may worsen mental health (19).

However, many previous studies have focused not only on quantitative indicators of outings (outing frequency, which means leaving their homes) but also on qualitative indicators of outings (outings with or without interpersonal interaction) as a condition for having hikikomori. The Japanese Cabinet Office included the quality of outings as a component of the definition of hikikomori. Specifically, occasional outings that do not require interpersonal interactions, such as to convenience stores or to pursue a hobby, are included in hikikomori (20, 21). Another study reported that behavioral characteristics related to the absence of social participation were associated only with outings that required interpersonal interactions but not with those that did not, such as walks and visits to convenience stores or supermarkets (22). Thus, some previous findings support the inclusion of both quantitative and qualitative indicators of outings as hikikomori components.

Furthermore, the proposed hikikomori definition uses outing frequency to define and determine severity, with outings of 4 days/week or more not meeting hikikomori criteria and outings of 1 day/week or less considered moderate. However, there is no clear theoretical or empirical evidence that one or 4 days/week is an appropriate criterion. It is also unclear how the proposed condition (that is, not including the qualitative aspects of the outing) differs in the extent of hikikomori compared to the existing condition (that is, including the qualitative aspects of the outing). This study aimed to fill those gaps and clarify the ambiguity in the relationship between hikikomori and frequency, a quantitative indicator of outings, and interpersonal interaction, a qualitative indicator of outings. More specifically, we provided the distribution of outing frequency using the hikikomori definition by the Ministry of Health, Labour and Welfare, which includes the qualitative aspect of outing as a condition, and showed the differences in individuals meeting the hikikomori condition. Furthermore, we examined whether the criteria of outing frequency (1 or 4 days per week) and qualitative indicators adequately predict hikikomori. People with hikikomori tend to avoid others, and the frequency and quality of outings may differ depending on the investigated sample. Therefore, this study used multiple samples for the analysis.

## 2. Methods

### 2.1. Participants

The data included three datasets: online self-rating, offline self-rating, and parent-rated samples. The parent rating dataset included both online and offline samples. These samples were analyzed separately for each dataset due to differences in the period during which the studies were performed and the data collection methods. Hikikomori individuals are less likely to have access to treatment and, therefore, may be less likely to have access to research. Thus, the parent-rated sample may have increased hikikomori severity compared to the self-rating sample. Data were collected in January 2020 for the online self-ratings sample, from November 2017 to February 2018 for the offline self-ratings sample, and from November 2016 to March 2017 for the parent-rated sample. Findings from the online self-rated sample data, focusing on the quality of life assessment, have been published in Nonaka and Sakai’s paper (18). Additionally, other findings from parent-rated sample data on the assessment of family interactions (23, 24), alongside socioeconomic factors and hikikomori (25), have been published. Some of the following published data in both studies were used to perform a secondary analysis: demographic variables, the number of days individuals went out per month, qualitative indicators of outings, and subjective social functioning impairment.

### 2.2. Instruments

Demographics. Age, gender, hikikomori duration, and the number of days individuals went out per month (0–30) were used in the analysis as quantitative indicators of outings (Supplementary material S1).

Qualitative indicators of outings. Three items, *“Going out freely*,” *“Going to places that require interpersonal interactions,”* and *“Going to places that do not require interpersonal interactions,”* represented the quality of outings, including interpersonal interaction. Each single item was rated on a four-point scale from 0 (*not true*) to 3 (*very true*), with higher scores indicating more truth. These items have been used in previous studies (17, 26).

Subjective social functioning impairment. Difficulties in social participation were assessed with one item rated on a 10-point scale from 1 (*never experience difficulty*) to 10 (*always experience difficulty*). This item has been used in previous studies (17, 26).

### 2.3. Procedure

The definition of hikikomori that was used in this study was provided by the Ministry of Health, Labor, and Welfare, specifically “a phenomenon characterized by a lack of social participation, which includes working, attending school, and socializing outside one’s home, and staying at home on most days except for solitary outings for over 6 months” (13). For the online sample, data were collected from people managed by an Internet research company with 2.2 million members in Japan. The company contacted the research panel managed by the company with a web link for the online questionnaire and its instructions. For the offline sample, data were collected in family associations on the topic of hikikomori with over 50 branches in regions throughout Japan. After obtaining consent for the research from the representatives of the family association, family association members were asked to participate in the research through their respective branches. Participants were asked to complete the questionnaires anonymously and send them by post. Participants were presented with the hikikomori definition in this study and asked if they or their child currently met that definition or if they had met it previously, and for how long. According to their self-reports on the questions, participants were divided into three groups: the hikikomori group for those who currently met the condition for over 6 months; the recovered group for those who did not currently meet the condition for over 6 months but did so previously; and control group for those who had never met the condition.

### 2.4. Data analysis

Jamovi version 2.3 and the modules (27–32) was used for data analysis. We obtained the outing frequency per week to show their distribution and classified them into the following seven categories: within 1 day, over 1 day and within 2 days, over 2 days and within 3 days, over 3 days and within 4 days, over 4 days and within 5 days, over 5 days and within 6 days, and over 6 days per week. Spearman’s correlation coefficients were obtained between the frequency of outings by decimal data and qualitative indicators of outings. We divided the participants based on their outing frequency per week into three levels according to the criteria of a previous study (15) 1 day or fewer (*low*), 4 days or more (*high*), and others (*medium*). Data with missing outing frequency were excluded from the analysis. Multinomial logistic regression analyzes were conducted on the online self-ratings and parent-rated samples with the hikikomori, recovered, and control groups as the dependent variables. Since the offline self-rating sample did not include the control group, a binomial logistic regression analysis was conducted for the hikikomori and recovered groups as the dependent variables. These were used to examine the suitability of quantitative or qualitative indicators of outings as a condition for hikikomori. The Variance Inflation Factor (VIF) was calculated to check for multicollinearity. The linearity relationship between continuous independent variables and the logit of the dependence and independence of errors were checked to confirm that these assumptions were met. There were no strongly influential outliers based on the detection of outliers using standardized residuals. Predictor variables were outing frequency (Low, Medium, High), “Going out freely,” “Going to places that require interpersonal interactions,” and “Going to places that do not require interpersonal interactions,” in addition to subjective social functioning impairment, the participant’s relationship (mother or father), and gender of hikikomori individuals. The reference levels for the categorical variables were the hikikomori group for the outcome variable, man for gender, and high (4–7 days) for outing frequency. Receiver operating characteristic (ROC) curves were used with data from all samples to identify cutoffs for outing frequency. In the parent-rated sample, Welch’s t-test was performed to examine the score differences for each variable between fathers and mothers of hikikomori individuals.

### 2.5. Ethical consideration

This study was approved by the local research ethics committee of Tokushima University (No. 102 and 133) and Tokyo Future University (No. 112). This research was performed anonymously and informed consent was obtained from the participants.

## 3. Results

### 3.1. Demographic characteristics

The online, offline, and parent-rated samples included 397, 72, and 784 individuals, respectively (Table 1). The participants resided in the following regions (online self-ratings; offline self-ratings; parent ratings, respectively): Hokkaido (4.5%; 4.2%; 4.6%), Tohoku (6.5%; 11.1%; 6.0%), Kanto (38.0%; 26.4%; 36.7%), Chubu (13.4%; 37.5%; 20.0%), Kinki (23.4%; 1.4%; 15.2%), Chugoku (3.5%; 2.8%; 4.1%), Shikoku (3.5%; 13.9%; 4.6%), and Kyushu (7.1%; 2.8%; 8.8%).

**Table 1:** Demographic characteristics of participants.

	Hikikomori group		Recovered group		Control group	
Self-reported (online sample)	*N*	(%)	*N*	(%)	*N*	(%)
Men	70	(70.7)	70	(70.0)	139	(70.2)
Women	29	(29.3)	30	(30.0)	59	(29.8)
	***M***	**(SD)**	***M***	**(SD)**	***M***	**(SD)**
Age	44.58	(9.69)	43.17	(9.55)	44.89	(9.80)
Hikikomori duration (months)	106.63	(87.92)	28.65	(40.45)	–	–
Self-reported (offline sample)	*N*	(%)	*N*	(%)	*N*	(%)
Men	28	(82.4)	30	(78.9)	–	–
Women	6	(17.6)	8	(21.1)	–	–
	***M***	**(SD)**	***M***	**(SD)**	***M***	**(SD)**
Age	36.06	(7.45)	36.61	(8.32)	–	–
Hikikomori duration (months)	163.97	(110.33)	90.42	(82.64)	–	–
Parent-reported (online/offline sample)	*N*	(%)	*N*	(%)	*N*	(%)
Men	202	(83.8)	59	(79.7)	383	(81.7)
Women	39	(16.2)	15	(20.3)	86	(18.3)
	***M***	**(SD)**	***M***	**(SD)**	***M***	**(SD)**
Age	32.63	(7.84)	33.18	(8.35)	32.83	(8.14)
Hikikomori duration (months)	128.47	(82.57)	73.87	(104.59)	–	–

### 3.2. Outing frequency tendency

Table 2 depicts outing frequency categories concerning online, offline, and parent-rated samples, 28.3, 35.3, and 47.7% had an outing frequency of 1 day/week or less, while 20.2, 20.6, and 14.5% had outing frequencies of four or more days/week in the hikikomori group.

**Table 2:** Outing frequency categories in each group.

Days	Hikikomori group (%)		Recovered group (%)		Control group (%)	
**Self-reported (online sample)**						
≤1	28	(28.3%)	10	(10.0%)	4	(2.0%)
1<, ≤2	23	(23.2%)	8	(8.0%)	15	(7.6%)
2<, ≤3	16	(16.2%)	7	(7.0%)	10	(5.1%)
3<, ≤4	12	(12.1%)	3	(3.0%)	9	(4.5%)
4<, ≤5	5	(5.1%)	19	(19.0%)	26	(13.1%)
5<, ≤6	5	(5.1%)	30	(30.0%)	36	(18.2%)
6<	10	(10.1%)	23	(23.0%)	98	(49.5%)
**Self-reported (offline sample)**						
≤1	12	(35.3%)	0	(0.0%)	–	
1<, ≤2	5	(14.7%)	3	(7.9%)	–	
2<, ≤3	4	(11.8%)	6	(15.8%)	–	
3<, ≤4	6	(17.6%)	5	(13.2%)	–	
4<, ≤5	3	(8.8%)	5	(13.2%)	–	
5<, ≤6	2	(5.9%)	8	(21.1%)	–	
6<	2	(5.9%)	11	(28.9%)	–	
**Parent-reported (online/offline sample)**						
≤1	115	(47.7%)	8	(10.8%)	12	(2.6%)
1<, ≤2	42	(17.4%)	8	(10.8%)	24	(5.1%)
2<, ≤3	32	(13.3%)	2	(2.7%)	23	(4.9%)
3<, ≤4	17	(7.1%)	7	(9.5%)	19	(4.1%)
4<, ≤5	13	(5.4%)	18	(24.3%)	55	(11.7%)
5<, ≤6	9	(3.7%)	12	(16.2%)	123	(26.2%)
6<	13	(5.4%)	19	(25.7%)	213	(45.4%)

### 3.3. Relationship between outing frequency and hikikomori tendency

Among the qualitative indicators of outings in hikikomori group, “Going out freely” consistently showed at least moderate positive correlations with outing frequency (Table 3), and the correlation between outing frequency and outing with or without interpersonal interaction was not consistent across sample types. However, a trend toward a higher correlation for outings without interpersonal interaction was detected than with it. Subjective social functioning impairment also showed no apparent trend of correlation. Positive moderate correlations between outing frequency and “Going out freely” were found in the Recovered group, while small or non-significant correlations were found in the Control group (Supplementary material S2). The correlations between subjective social functioning impairment and “Going to places that require interpersonal interaction” were small or nonsignificant in the Recovered group but moderately negatively correlated in the Control group.

**Table 3:** Correlation of each variable with outing frequency or subjective social functioning impairment for the Hikikomori group.

	Self-reported (online sample)			Self-reported (offline sample)			Parent-reported (online/offline sample)		
	Spearman’s *ρ*	*p*	*n*	Spearman’s *ρ*	*p*	*n*	Spearman’s *ρ*	*p*	*n*
**with outing frequency**									
Going out freely	0.497	< 0.001	99	0.462	0.006	34	0.738	< 0.001	236
Going to places that require interpersonal interactions	0.203	0.043	99	0.089	0.616	34	0.342	< 0.001	234
Going to places that do not require interpersonal interactions	0.384	< 0.001	99	0.193	0.282	33	0.647	< 0.001	233
Subjective social functioning impairment	−0.088	0.387	99	−0.271	0.121	34	−0.166	0.010	239
**with subjective social functioning impairment**									
Going out freely	−0.216	0.032	99	−0.116	0.514	34	−0.163	0.012	236
Going to places that require interpersonal interactions	−0.089	0.383	99	−0.221	0.209	34	−0.257	< 0.001	234
Going to places that do not require interpersonal interactions	0.029	0.777	99	0.082	0.649	33	−0.050	0.451	233

### 3.4. Association of hikikomori with outing-related indicators and subjective social impairment

Logistic regression analysis showed that the model was significant for the online self-rating (McFadden’s *R^2^* = 0.23, *p* < 0.001), offline self-rating (McFadden’s *R^2^* = 0.42, *p* < 0.001), and parent-rated (McFadden’s *R^2^* = 0.59, *p* < 0.001) samples. The hikikomori group was associated with a lower frequency of outings (Low vs. High, Medium vs. High), lower outings with interpersonal interaction, and higher subjective social impairment compared to the recovered and control groups (Table 4). This trend was consistent across all three samples. In contrast, going out freely and outings without interpersonal interaction did not predict whether one was a hikikomori. VIF ranged from 1.02–1.30 for the online self-rating sample, 1.01–1.26 for the offline self-rating sample, and 1.03–1.32 for the parent-rated sample, all the values being lower than 2.

**Table 4:** Multinomial logistic regression analyzes for groups on hikikomori.

Model Coefficients -Group										
		Self-reported (online sample)			Self-reported (offline sample)			Parent-reported (online/offline sample)		
Group	Predictor	Estimate	(95% CI)	*p*	Estimate	(95% CI)	*p*	Estimate	(95% CI)	*p*
**Recovered Group (Reference level: Hikikomori Group)**										
	Intercept	2.17	(0.64, 3.69)	0.005	2.42	(−1.86, 6.70)	0.27	0.55	(−1.14, 2.24)	0.52
	Gender (Reference level: Man)									
	Woman	0.35	(−0.38, 1.07)	0.35	0.21	(−1.69, 2.10)	0.83	0.26	(−0.61, 1.13)	0.56
	Participants (Reference level: Mother)									
	Father							0.47	(−0.26, 1.20)	0.20
	Outing frequency (Reference level: High [4≤ days])									
	Low (≤1 day)	−2.02	(−3.03, −1.02)	< 0.001	−19.05	(−3451.14, 3413.04)	0.99	−2.18	(−3.24, −1.11)	< 0.001
	Medium (1–4 days)	−2.28	(−3.04, −1.52)	< 0.001	−1.50	(−2.96, −0.04)	0.04	−1.76	(−2.58, −0.94)	< 0.001
	(1)	0.02	(−0.40, 0.43)	0.94	−0.30	(−1.27, 0.66)	0.54	−0.03	(−0.44, 0.37)	0.87
	(2)	0.42	(0.06, 0.77)	0.02	1.14	(0.14, 2.15)	0.03	1.18	(0.79, 1.58)	< 0.001
	(3)	−0.11	(−0.47, 0.26)	0.57	−0.04	(−1.04, 0.96)	0.94	−0.30	(−0.68, 0.08)	0.12
	(4)	−0.21	(−0.36, −0.06)	0.006	−0.38	(−0.75, −0.01)	0.04	−0.25	(−0.40, −0.09)	0.001
Control Group (Reference level: Hikikomori Group)^*^										
	Intercept	3.32	(1.81, 4.84)	< 0.001				3.31	(1.74, 4.88)	< 0.001
	Gender (Reference level: Man)									
	Woman	0.33	(−0.39, 1.05)	0.36				0.44	(−0.44, 1.32)	0.33
	Participants (Reference level: Mother)									
	Father							1.72	(1.00, 2.45)	< 0.001
	Outing frequency (Reference level: High [4≤ days])									
	Low (≤1 day)	−3.24	(−4.50, −1.97)	< 0.001				−2.93	(−4.13, −1.74)	< 0.001
	Medium (1–4 days)	−2.28	(−3.00, −1.57)	< 0.001				−1.96	(−2.77, −1.16)	< 0.001
	(1)	0.18	(−0.23, 0.60)	0.39				0.42	(−0.02, 0.85)	0.06
	(2)	0.59	(0.23, 0.94)	0.001				0.73	(0.32, 1.14)	< 0.001
	(3)	−0.16	(−0.52, 0.20)	0.40				−0.28	(−0.66, 0.11)	0.17
	(4)	−0.40	(−0.54, −0.25)	< 0.001				−0.73	(−0.89, −0.58)	< 0.001

CI: Confidence interval, (1) Going out freely, (2) Going to places that require interpersonal interactions, (3) Going to places that do not require interpersonal interactions, and (4) Subjective impairment of social functioning, ^*^Binomial logistic regression analysis was performed on the self-reported (offline sample).

The Area Under the Curve (AUC) was 0.874 between the hikikomori and control groups and 0.797 between the hikikomori and recovered groups. The cut-off point for the highest Youden’s index was 20 days (Sensitivity 82.16%, Specificity 83.96%, Youden’s index 0.661) outside the home per month between the hikikomori and control groups, followed by 18 days (Sensitivity 82.61%, Specificity 83.42%, Youden’s index 0.660). Between the hikikomori group and the recovered group, the cut-off point for the highest Youden’s index was 17 days (Sensitivity 68.87%, Specificity 83.16%, Youden’s index 0.520) outside the home per month, followed by 18 days (Sensitivity 68.40%, Specificity 83.42%, Youden’s index 0.518). This means that the cut-off point for identifying a hikikomori was 4.0–4.7 days outside the home per week.

In the hikikomori group for parent rating, there were no significant score differences for each variable between mothers and fathers (social functioning impairment: *t* (126.95) = 1.60, *p* = 0.11, *d* = 0.23; outing frequency: *t* (138.42) = 0.99, *p* = 0.33, *d* = 0.14; Going out freely: *t* (121.79) = 0.95, *p* = 0.35, *d* = 0.14; Going to places that require interpersonal interactions: *t* (122.66) = 0.11, *p* = 0.92, *d* = 0.02; Going to places that do not require interpersonal interactions: *t* (118.20) = 0.12, *p* = 0.91, *d* = 0.02).

## 4. Discussion

This study aimed to clarify the relationship between hikikomori, outing frequency, and the qualitative aspects of outings. As a result, the outing frequency of individuals with hikikomori compared to those in recovery and those who did not experience hikikomori was quite distinctive, showing an overall trend toward infrequent outings. The AUC showed moderate accuracy, and the cut-off points supported the criteria for the number of days outside the home proposed in previous studies (14, 15).

Regarding the criteria (15) of going out four or more days/week, the frequency distribution of outings showed that 14.5 to 20.6% of those included by the previous definition would not be considered to have hikikomori. Correlations between frequency of outings and interpersonal interaction-related indicators in the hikikomori group were inconsistent across sample types, with the frequency of outings correlating moderately or less with outings with interpersonal interaction and weak to high correlations with outings without interpersonal interaction. Therefore, the quantity and quality of outings would reflect different aspects. Additionally, the frequency of outings showed a weak or less correlation with subjective social functioning impairment. The weak association between going out freely and outing frequency only for the Control group may indicate that they “have to go out” for work and therefore go out “not freely” more often. The fact that only the control group showed consistently negative correlations between subjective social functioning impairment and outings with interpersonal interaction might reflect that hikikomori individuals rarely go on outings with interpersonal interaction.

An essential finding of this study is the difference that outings with interpersonal interaction predicted hikikomori, but those without interaction did not. This difference supports Sakai et al.’s study (22) and another report (33) wherein interpersonal relationships were strongly associated with hikikomori. Although lack of social interaction was identified as an additional characteristic of hikikomori in Kato et al.’s study (15), the lack of social interaction should be characterized as one of the hikikomori conditions. The frequency of outings and social functioning impairment also predicted the extent of hikikomori. This finding supports Kato et al.’s suggestion (15). The result that frequency of outings also predicted comparison between hikikomori and recovered individuals indicates that frequency of outings is one of the components of hikikomori status. The results support previous studies that fewer outing frequencies are associated with hikikomori (16–18). Furthermore, this study provided findings that partially support the recently proposed outing frequency condition for hikikomori (14, 15) but that the association between outing behavior and hikikomori differed depending on whether it involved interpersonal interaction or not. Thus, outing frequency may have significant interpretability, although it cannot wholly discriminate between those who have and do not have hikikomori, compared to the previous definition. There is a report that people with hikikomori for over 6 months are associated with less face-to-face expressing distress rather than less face-to-face contacting with others (3). Considering these results, focusing not only on the frequency of outings but also on the purpose and function of outings may help resolve the criterion problem, excluding some of those who are thought to have hikikomori.

As initially expected, those with hikikomori tended to differ in their outing frequency depending on the sample type. The highest percentage of one or fewer outing days/week was seen in the parent-rated sample (47.7%), followed by the self-rated offline sample (35.3%) and the self-rated online sample (28.3%). While the parent-rated sample may have a stronger hikikomori tendency than the self-rated sample, the online sample may have a weaker hikikomori tendency than the offline samples. These trends may reflect research accessibility, similar to treatment accessibility. On the other hand, the results of the percentages of outings exceeding 4 days/week were similar across online and offline self-ratings. This result suggests that the differences in outing frequency by sample type may be more marked among those with relatively more severe hikikomori, that is, who do not go out very often.

### 4.1. Limitations

This study used a cross-sectional design to determine outing frequency, and findings may have been biased by memory. Therefore, prospective longitudinal studies will be needed in the future. Kato et al. (14) also included significant functional impairment or distress as a condition for hikikomori, and there is not necessarily any consensus on this condition as a definition of hikikomori. The results showed a weak or no correlation between outing frequency and subjective social functioning impairment. Future work is needed to clarify the influence of significant functional impairment or distress on the extent of hikikomori. There is still no gold standard tool for assessing qualitative aspects of outings. Even though we used items that have been implemented in several previous studies, the lack of an appropriately reliable and validated instrument is a severe limitation of this study. Although data from multiple collection methods were analyzed to reduce sample bias, the sample size was insufficient in the self-reported offline sample, and no recovered individuals indicated an outing frequency of 1 day or less per week. It is thus necessary to carefully interpret the influence that this had on the results of the logistic regression analysis.

## 5. Conclusion

The study found that not including low social interaction in the hikikomori condition may result in 14–20% fewer individuals meeting the hikikomori definition compared to including low social interaction in the condition. Furthermore, outings with interpersonal interaction, outing frequency, and subjective social functioning impairment predicted the extent of hikikomori. For integration with previous findings, the results showed that in defining hikikomori and determining its severity, in addition to outing frequency, which is a quantitative indicator, it is necessary to identify whether or not the outing involves interpersonal interaction, which is a qualitative indicator.

## Data availability statement

The raw data supporting the conclusions of this article will be made available by the authors, without undue reservation.

## Ethics statement

The studies involving human participants were reviewed and approved by Research Ethics and Misconduct Prevention Committee, Tokyo Future University Research Ethics committee of Graduate school of Integrated Arts and Sciences, Tokushima University. Written informed consent for participation was not required for this study in accordance with the national legislation and the institutional requirements.

## Author contributions

SN: conceptualization, data curation, formal analysis, funding acquisition, investigation, methodology, project administration, resources, visualization, writing-original draft, and writing-review and editing. MS: funding acquisition, investigation, methodology, supervision, and writing-review and editing. All authors contributed to the article and approved the submitted version.

## Funding

This work was supported by JSPS KAKENHI grants (Grant numbers: 16J10405, 25780416, 20K14199, 21H00951) and by the grants from the Ministry of Health, Labour and Welfare of Japan. The funding source had no role in the study design; the collection, analysis, and interpretation of data, the writing of the report, or the decision to submit the article for publication.

## Conflict of interest

The authors declare that the research was conducted in the absence of any commercial or financial relationships that could be construed as a potential conflict of interest.

## Publisher’s note

All claims expressed in this article are solely those of the authors and do not necessarily represent those of their affiliated organizations, or those of the publisher, the editors and the reviewers. Any product that may be evaluated in this article, or claim that may be made by its manufacturer, is not guaranteed or endorsed by the publisher.
