# Supplementary material for: The suitability of outing frequency as a definition of hikikomori (prolonged social withdrawal)
Source: Front Psychiatry. 2023 Mar 16;14:1027498. doi: 10.3389/fpsyt.2023.1027498 (PMC10061135; doi:10.3389/fpsyt.2023.1027498)
Supplement: Supplementary file 1 [file Data_Sheet_1.pdf]

Supplementary material 1

The questionnaire items are in Japanese, which the authors translated preliminarily into English.

A. Online self-rating questionnaire (List of items used in the analysis)

Hikikomori is "a phenomenon characterized by a lack of social participation, which includes working, attending school, and socializing outside one's home, and staying at home on most days except for solitary outings" in this study.

1. Have you experienced Hikikomori?

   ・I am currently a hikikomori.
   ・I am not currently a hikikomori, but I have been a hikikomori in the past.
   ・I have never been a hikikomori.

2. According to the example below, please answer the duration of your hikikomori.

   (Example) If you were in hikikomori for one year and six months from age 19 and five years and three months from age 24
   1st time: from the age of (19), (1) year and (6) months
   2nd time: from the age of (24), (5) years and (3) months

   1st time: from the age of ( ), ( ) year and ( ) months
   2nd time: from the age of ( ), ( ) years and ( ) months
   3rd time: from the age of ( ), ( ) years and ( ) months

3. Please answer the average number of days you went out per month.

   → Average ( ) days per month

4. The following questions ask about your condition during the last two weeks. Please select one that applies to each.

   ・ Going out freely: 0 (not true), 1 (not very true), 2 (a little true), 3 (very true)
   ・ Going to places that require interpersonal interactions: 0 (not true), 1 (not

very true), 2 (a little true), 3 (very true)

- Going to places that do not require interpersonal interactions: 0 (not true), 1 (not very true), 2 (a little true), 3 (very true)

5. Do you feel difficulties in social participation?

Please select the number that best describes your feeling from 1 (never experience difficulty) to 10 (always experience difficulty).

B. Offline self-rating questionnaire (List of items used in the analysis)

Hikikomori is "a phenomenon characterized by a lack of social participation, which includes working, attending school, and socializing outside one's home, and staying at home on most days except for solitary outings" in this study.

1a. Are you currently a hikikomori? → a. Yes b. No
1b. Have you ever been a hikikomori in the past? → a. Yes b. No

2. According to the example below, please answer the duration of your hikikomori.

(Example) If you were in hikikomori for one year and six months from age 19 and five years and three months from age 24
1st time: from the age of (19), (1) year and (6) months
2nd time: from the age of (24), (5) years and (3) months

1st time: from the age of ( ), ( ) year and ( ) months
2nd time: from the age of ( ), ( ) years and ( ) months
3rd time: from the age of ( ), ( ) years and ( ) months

3. Please answer the average number of days you went out per month.

→ Average ( ) days per month

4. The following questions ask about your condition during the last two weeks. Please select one that applies to each.

- Going out freely: 0 (not true), 1 (not very true), 2 (a little true), 3 (very true)
- Going to places that require interpersonal interactions: 0 (not true), 1 (not very true), 2 (a little true), 3 (very true)
- Going to places that do not require interpersonal interactions: 0 (not true), 1 (not very true), 2 (a little true), 3 (very true)

5. Do you feel difficulties in social participation?
Please select the number that best describes your feeling from 1 (never experience difficulty) to 10 (always experience difficulty).

C1. Online parent rating questionnaire (List of items used in the analysis)

If you have more than one child, please select one freely and answer the question below. If you have a child who has experienced hikikomori, please select the child and answer the question.

Hikikomori is "a phenomenon characterized by a lack of social participation, which includes working, attending school, and socializing outside one's home, and staying at home on most days except for solitary outings" in this study.

1. Has the child experienced Hikikomori?
   - The child is currently a hikikomori.
   - The child is not currently a hikikomori, but the child has been a hikikomori in the past.
   - The child has never been a hikikomori.

2. According to the example below, please answer the duration of the child's hikikomori.

   (Example) If the child was in hikikomori for one year and six months from age 19 and five years and three months from age 24
   1st time: from the age of (19), (1) year and (6) months
   2nd time: from the age of (24), (5) years and (3) months

   1st time: from the age of ( ), ( ) year and ( ) months
   2nd time: from the age of ( ), ( ) years and ( ) months
   3rd time: from the age of ( ), ( ) years and ( ) months

3. Please answer the average number of days the child went out per month.

   → Average ( ) days per month

4. The following questions ask about the child's condition during the last two weeks (in case of separation, as recently as known). Please select one that applies to each.
   - Going out freely: 0 (not true), 1 (not very true), 2 (a little true), 3 (very true)
   - Going to places that require interpersonal interactions: 0 (not true), 1 (not

very true), 2 (a little true), 3 (very true)

- Going to places that do not require interpersonal interactions: 0 (not true), 1 (not very true), 2 (a little true), 3 (very true)

5. Do you think the child feels difficulties in social participation?

Please select the number that best describes the child's feeling from 1 (never experience difficulty) to 10 (always experience difficulty).

C2. Offline parent rating questionnaire (List of items used in the analysis)

If you have more than one child, please select one freely and answer the question below. If you have a child who has experienced hikikomori, please select the child and answer the question.

Hikikomori is "a phenomenon characterized by a lack of social participation, which includes working, attending school, and socializing outside one's home, and staying at home on most days except for solitary outings" in this study.

1a. Is the child currently a hikikomori? → a. Yes b. No
1b. Has the child ever been a hikikomori in the past? → a. Yes b. No

2. According to the example below, please answer the duration of the child's hikikomori.

(Example) If the child was in hikikomori for one year and six months from age 19 and five years and three months from age 24
1st time: from the age of (19), (1) year and (6) months
2nd time: from the age of (24), (5) years and (3) months

1st time: from the age of ( ), ( ) year and ( ) months
2nd time: from the age of ( ), ( ) years and ( ) months
3rd time: from the age of ( ), ( ) years and ( ) months

3. Please answer the average number of days the child went out per month.

→ Average ( ) days per month

4. The following questions ask about the child's condition during the last two weeks (in case of separation, as recently as known). Please select one that applies to each.

- Going out freely: 0 (not true), 1 (not very true), 2 (a little true), 3 (very true)
- Going to places that require interpersonal interactions: 0 (not true), 1 (not very true), 2 (a little true), 3 (very true)
- Going to places that do not require interpersonal interactions: 0 (not true), 1 (not very true), 2 (a little true), 3 (very true)

5. Do you think the child feels difficulties in social participation?
Please select the number that best describes the child's feeling from 1 (never experience difficulty) to 10 (always experience difficulty).
