# Supplementary material for: The suitability of outing frequency as a definition of hikikomori (prolonged social withdrawal)
Source: Front Psychiatry. 2023 Mar 16;14:1027498. doi: 10.3389/fpsyt.2023.1027498 (PMC10061135; doi:10.3389/fpsyt.2023.1027498)
Supplement: Supplementary file 2 [file Data_Sheet_2.docx]

**Supplementary material 2a**.

Correlation of each variable with outing frequency or subjective social functioning impairment for the Recovered group

|  |  | Self-reported (online sample) | | | Self-reported (offline sample) | | | Parent-reported (online/offline sample) | | |
| --- | --- | --- | --- | --- | --- | --- | --- | --- | --- | --- |
|  |  | Spearman's ρ | *p* | *n* | Spearman's ρ | *p* | *n* | Spearman's ρ | *p* | *n* |
| with outing frequency | | |  |  |  |  |  |  |  |  |
|  | Going out freely | 0.382 | < .001 | 100 | 0.360 | 0.026 | 38 | 0.312 | 0.008 | 72 |
|  | Going to places that require interpersonal interactions | 0.277 | 0.005 | 100 | 0.103 | 0.540 | 38 | 0.365 | 0.002 | 72 |
|  | Going to places that do not require interpersonal interactions | 0.122 | 0.228 | 100 | 0.287 | 0.081 | 38 | -0.094 | 0.433 | 71 |
|  | Subjective social functioning impairment | 0.006 | 0.951 | 100 | -0.224 | 0.189 | 36 | -0.277 | 0.017 | 74 |
| with subjective social functioning impairment | | | |  |  |  |  |  |  |  |
|  | Going out freely | -0.064 | 0.526 | 100 | -0.177 | 0.301 | 36 | -0.377 | 0.001 | 72 |
|  | Going to places that require interpersonal interactions | -0.286 | 0.004 | 100 | -0.232 | 0.173 | 36 | -0.223 | 0.060 | 72 |
|  | Going to places that do not require interpersonal interactions | 0.072 | 0.477 | 100 | -0.276 | 0.103 | 36 | 0.327 | 0.005 | 71 |

**Supplementary material 2b**.

Correlation of each variable with outing frequency or subjective social functioning impairment for the Control group

|  |  | Self-reported (online sample) | | | Parent-reported (online/offline sample) | | |
| --- | --- | --- | --- | --- | --- | --- | --- |
|  |  | Spearman's ρ | *p* | *n* | Spearman's ρ | *p* | *n* |
| with outing frequency | | |  |  |  |  |  |
|  | Going out freely | 0.120 | 0.091 | 198 | 0.190 | < .001 | 468 |
|  | Going to places that require interpersonal interactions | 0.191 | 0.007 | 198 | 0.215 | < .001 | 468 |
|  | Going to places that do not require interpersonal interactions | -0.046 | 0.522 | 198 | 0.012 | 0.794 | 468 |
|  | Subjective social functioning impairment | -0.148 | 0.038 | 198 | -0.152 | < .001 | 469 |
| with subjective social functioning impairment | | | |  |  |  |  |
|  | Going out freely | -0.214 | 0.002 | 198 | -0.308 | < .001 | 468 |
|  | Going to places that require interpersonal interactions | -0.316 | < .001 | 198 | -0.423 | < .001 | 468 |
|  | Going to places that do not require interpersonal interactions | 0.007 | 0.925 | 198 | 0.063 | 0.173 | 468 |
